# Effects of impurities on membrane-protein crystallization in different systems

**DOI:** 10.1107/S0907444909029163

**Published:** 2009-09-16

**Authors:** Christopher A. Kors, Ellen Wallace, Douglas R. Davies, Liang Li, Philip D. Laible, Peter Nollert

**Affiliations:** a Biosciences Division, Argonne National Laboratory, 9700 South Cass Avenue, Argonne, IL 60439, USA; b deCODE biostructures, 7869 NE Day Road West, Bainbridge Island, WA 98110, USA; c Department of Chemistry and Institute for Biophysical Dynamics, University of Chicago, 929 East 57th Street, Chicago, IL 60637, USA

**Keywords:** membrane proteins, impurities, crystallization

## Abstract

When starting a protein-crystallization project, scientists are faced with several unknowns. Amongst them are these questions: (i) is the purity of the starting material sufficient? and (ii) which type of crystallization experiment is the most promising to conduct? The difficulty in purifying active membrane-protein samples for crystallization trials and the high costs associated with producing such samples require an extremely pragmatic approach. Additionally, practical guidelines are needed to increase the efficiency of membrane-protein crystallization. In order to address these conundrums, the effects of commonly encountered impurities on various membrane-protein crystallization regimes have been investigated and it was found that the lipidic cubic phase (LCP) based crystallization methodology is more robust than crystallization in detergent environments using vapor diffusion or microbatch approaches in its ability to tolerate contamination in the forms of protein, lipid or other general membrane components. LCP-based crystallizations produced crystals of the photosynthetic reaction center (RC) of *Rhodobacter sphaeroides* from samples with substantial levels of residual impurities. Crystals were obtained with protein contamination levels of up to 50% and the addition of lipid material and membrane fragments to pure samples of RC had little effect on the number or on the quality of crystals obtained in LCP-based crystallization screens. If generally applicable, this tolerance for impurities may avoid the need for samples of ultrahigh purity when undertaking initial crystallization screening trials to determine preliminary crystallization conditions that can be optimized for a given target protein.

## Abstract 2

The effects of commonly encountered impurities on various membrane-protein crystallization regimes are investigated and it is found that the lipidic cubic phase crystallization methodology is the most robust, tolerating protein contamination levels of up to 50%, with little effect on crystal quality. If generally applicable, this tolerance may be exploited (i) in initial crystallization trials to determine the ‘crystallizability’ of a given membrane-protein and (ii) to subject partially pure membrane-protein samples to crystallization trials.

## 1. Introduction

The relatively low number of currently available X-ray crystallographic membrane-protein structures compared with those of soluble proteins points to the need for better methods for membrane-protein crystallization (von Heijne, 2007 ▶). Most of the atomic level details can be directly accredited to the generation of protein crystals suitable for structure determination by X-ray crystallography.

Membrane proteins are comprised of hydrophobic and hydrophilic regions, rendering them soluble in the cell mem­brane and in artificial amphiphile structures such as lipid bilayers and detergent micelles. For purification and subsequent biochemical and biophysical analyses, the extraction of membrane proteins from their native membrane is achieved through solubilization with a detergent, forming protein–detergent complexes (PDCs; le Maire *et al.*, 2000 ▶; Moller & le Maire, 1993 ▶). The properties of a particular solubilizing detergent affect the extraction yield, protein purity, type and quantity of co-extracted lipids, as well as the functional and structural integrity of the protein (Garavito & Ferguson-Miller, 2001 ▶; Privé, 2007 ▶). It is important that the artificial microenvironment is suitable to support the native conformation of the membrane protein and allows productive protein–protein interactions during crystallization and prevents protein precipitation and phase separation. Current standard laboratory practice calls for the purification of a target protein sample to usually better than ∼90% as judged by SDS–PAGE prior to submitting samples to crystallization trials. However, it is unknown whether a protein sample is truly ‘pure enough’ to produce crystals. With regard to the crystallization of soluble proteins, a study (Geerlof *et al.*, 2006 ▶) backed this laboratory practice, showing that proteins of unknown structure with purity levels of >95% (as determined by SDS–PAGE) yielded crystals in 59% of all instances, whereas samples that were <95% pure yielded crystals with a success rate of only 37%. A different study investigating the effects of macromolecular impurities on the crystallization of eubacterial aspartyl-tRNA synthetase, either by vapor diffusion or in capillaries, called for purity-level requirements of ∼99% for the production of high-quality crystals (Moreno *et al.*, 2005 ▶). Findings such as these point towards protein purity being a dominant contributor to failed crystallization attempts and have formed more-or-less unsubstantiated guidelines for purity requirements in membrane-protein crystallization trials.

With respect to lipid content, the guidelines for membrane-protein crystallization have changed over time. At the dawn of membrane-protein crystallization, it was believed to be of the utmost importance to remove as many endogenous lipids from the extracted protein molecules as possible. However, results over the years  have changed this perception (De Foresta *et al.*, 1994 ▶; Garavito & Ferguson-Miller, 2001 ▶; Garavito *et al.*, 1996 ▶; Haneskog *et al.*, 1996 ▶; Kragh-Hansen *et al.*, 1998 ▶; Lund *et al.*, 1989 ▶). The proper functioning of membrane proteins in the complex environment of a biological lipid bilayer (White & Wimley, 1999 ▶) may require specific lipid–membrane protein interactions and indeed lipids have been directly observed in several membrane-protein crystal structures (Ferguson *et al.*, 2000 ▶; Jones, 2007 ▶; Camara-Artigas *et al.*, 2002 ▶; McAuley *et al.*, 1999 ▶; Roszak *et al.*, 2007 ▶; Zhang *et al.*, 2003 ▶; Belrhali *et al.*, 1999 ▶; Ferguson *et al.*, 1998 ▶; Harrenga & Michel, 1999 ▶; Nollert, 2005 ▶; Tsukihara *et al.*, 1996 ▶; Jordan *et al.*, 2001 ▶). The supplementation of solubilized membrane-protein samples with lipids after their purification has improved or was required in the procedure to crystallize several membrane proteins (Guan *et al.*, 2006 ▶; Newman *et al.*, 1981 ▶; Toyoshima *et al.*, 2000 ▶).

For the growth of membrane-protein crystals of sufficient quality for X-ray diffraction experiments, protein particles must associate in an amphiphilic environment that leads to the formation of an ordered crystalline structure, as opposed to a non-ordered aggregated material (Nollert, 2005 ▶). This is practically pursued by testing crystallization conditions in crystallization trials, the latter of which are usually conducted in time-consuming trial-and-error experiments in which hundreds or even thousands of crystallization cocktails are sampled (Chang *et al.*, 1998 ▶; Dahl *et al.*, 2004 ▶). In these trials most experiments produce aggregated material, rarely pro­viding clues for further experiments. The initial discovery of a crystallization ‘hit’ for a given membrane protein, however, is an important milestone since these crystals demonstrate that many of the experimental parameters required for crystal growth have been identified and optimized parameters may be within short reach. Even the appearance of tiny or poorly diffracting crystals confirms that the protein under investigation can indeed form crystals (is ‘crystallizable’) and, provided that the protein sample and crystallization parameters can be improved, better crystals may eventually form. In addition, the crystalline material itself can be utilized as seeds in further crystallization experiments (Bergfors, 1999 ▶).

Conversely, the formation of unproductive aggregates may arise for a number of reasons including poor quality or purity of the protein sample, insufficient chemical composition of the crystallization cocktail or inadequate crystallization kinetics. Most researchers eventually call into question the purity of their sample or their choice of purification detergent. Indeed, protein-sample purity is important for successful nucleation, growth, and affects crystal quality since impurities cause undesirable interactions on the surface of growing crystals (Anderson *et al.*, 1988 ▶; Caylor *et al.*, 1999 ▶; Kurihara *et al.*, 1999 ▶; Plomp *et al.*, 2003 ▶; Van der Laan *et al.*, 1989 ▶; Vekilov & Rosenberger, 1996 ▶). Impurities are often associated with ‘step pinning’, where they adsorb to the surface of a growing crystal and impede the addition of desired components (Land *et al.*, 1999 ▶; McPherson *et al.*, 1996 ▶; Plomp *et al.*, 2003 ▶; Sangwal, 1996 ▶; van Enckevort *et al.*, 1996 ▶).

The sitting-drop vapour-diffusion technique produced the crystals used to solve the first known structure of a membrane protein (Deisenhofer *et al.*, 1984 ▶) and has long been most frequently employed for membrane-protein crystallization. The preparation of such crystallization experiments involves combining solutions of salts and/or polyethylene glycol (PEG) with a protein sample (small amphiphiles such as heptane-1,2,3-triol can also be supplemented), causing the protein to become supersaturated, which is aided by concomitant con­trolled dehydration. If conditions are favorable, the growth of structured and highly ordered protein crystals ensues after the formation of stable nuclei (Caffrey, 2003 ▶; Wiener, 2004 ▶).

Over a decade later, the crystallization of bacterio­rhodopsin within a lipidic cubic phase (LCP) matrix was first described (Landau & Rosenbusch, 1996 ▶). This crystallization methodology involves two simple steps. At first, the protein solution is mixed with a lipid, for example monoolein. In this material, the lipid self-assembles into a continuous bilayer, for example a lipidic cubic phase, containing the membrane protein. In a second step, crystallization is initiated by adding a crystallization-inducing reagent to the lipid material. The mechanism of crystallization from LCP is not fully understood (Nollert *et al.*, 2001 ▶), but is likely to involve diffusion within the bilayer and local concentration of the protein and restructuring of the lipid mesophase to a lamellar arrangement where crystal growth occurs through stacked bilayers (Caffrey, 2008 ▶; Grabe *et al.*, 2003 ▶). While the actual format has changed (Cherezov & Caffrey, 2006 ▶; Cherezov *et al.*, 2004 ▶; Nollert, 2002 ▶), this methodology has been key to the crystallization and subsequent structure determination of a number of membrane proteins, most prominently that of the β-adrenenergic receptor (Cherezov *et al.*, 2007 ▶).

Interestingly, the crystallization of the bacterial photosynthetic reaction center from *Rhodopseudomonas viridis* in a monoolein-based matrix (Katona *et al.*, 2003 ▶) starts out in a bona fide LCP and, depending on the duration of the crystallization experiment, the lipid matrix loses viscosity but remains transparent and nonbirefringent. Lipidic cubic phases are typically highly viscous, however, and in the absence of further characterization of the exact nature of the materials when crystals form, such host matrices will be referred to as ‘lipid mesophases’.

In order to minimize protein-sample consumption in crystallization trials, a plug-based crystallization technique akin to miniaturized microbatch setups (Chayen, 1992 ▶) has been developed. Unlike in vapor diffusion, the concentration of each component in the crystallization experiment, once set up, remains constant. Porin from *Rhodobacter capsulatus* and the photosynthetic reaction center (RC) from *Rhodopseudomonas viridis* have been crystallized using this microfluidic approach (Li *et al.*, 2006 ▶). Crystallization-inducing agent(s), buffer and protein are combined in microchannels made from polydimethylsiloxane (PDMS) and simultaneously form ∼10–­20 nl crystallization experiments in the form of ‘plugs’ carried by immiscible fluorinated oil (Zheng *et al.*, 2005 ▶).

The goal of this study was to compare (i) vapor-diffusion, (ii) plug-based and (iii) lipidic cubic phase-based crystallization approaches with regard to the sensitivity of crystallization success to sample purity. These experiments were designed in order to develop best practices in membrane-protein crystallization projects. The bacterial photosynthetic reaction center (RC; Allen *et al.*, 1987 ▶; Arnoux *et al.*, 1995 ▶; Chang *et al.*, 1991 ▶; Deisenhofer *et al.*, 1985 ▶; Ermler *et al.*, 1994 ▶; McAuley-Hecht *et al.*, 1998 ▶; Stowell *et al.*, 1997 ▶) was employed as a model membrane protein.

This study shows that the LCP crystallization method produces crystals from RC samples with substantial impurity levels and in this respect outperforms all other tested methods. If this finding is applicable to many other membrane proteins, then its tolerance for impurities predestines the LCP crystallization method as an effective tool for initial crystallization screening trials.

## 2. Materials and methods

### 2.1. Preparation of RC samples

*Rhodobacter sphaeroides* strains expressing recombinant polyhistidine-tagged RCs [C-terminal tag (7 × CAC) on the *M* subunit; GenBank Accession No. K00827; Pokkuluri *et al.*, 2002 ▶] were cultured in YCC medium (Taguchi *et al.*, 1992 ▶) for 2–3 d in 2.8 l Fernbach flasks (2 l per flask). Cells were harvested at 12 500*g*. The pellets were combined and washed in buffer 1 (10 m*M* Tris pH 7.8, 10 m*M* NaCl). The cell pellets were resuspended in buffer 1 and lysed by sonication and three serial passages through a microfluidizer (Model M-110L, Microfluidics, Newton, Massachusetts, USA). Unbroken cells and debris were removed by centrifugation at 22 000*g* for 15 min at 277 K. Membranes were pelleted by ultracentrifugation of the supernatant at 245 000*g* for 120 min at 277 K. Membrane pellets were weighed and resuspended in buffer 1 at 12.5 ml g^−1^ using a tissue homogenizer. The proteins embedded within these homogenized membranes were then solubilized by incubation for 2–3 min at 310 K (with stirring, in darkness) in 1%(*w*/*v*) *N*,*N*-dimethyldodecylamine-*N*-oxide (LDAO; Sigma–Aldrich, St Louis, Missouri, USA; CMC 0.023% as per Hermann, 1962 ▶).

Membrane debris was removed by ultracentrifugation of the suspension at 245 000*g* for 120 min at 277 K. The supernatant was then filtered (0.45 µm) prior to protein purification *via* one of the two following methods.

(i) The sample of lowest purity level (*A*_280_/*A*_800_ = 2.4) was prepared using customized automated scripts (adapted from Kirmaier *et al.*, 2005 ▶) on an ÄKTA FPLC (GE Healthcare, Piscataway, New Jersey, USA), employing incomplete column-washing steps. In this method, the supernatants were passed twice over a 5 ml HiTrap Chelating HP Column (GE Health­care) prepared with 0.1 *M* NiSO_4_. The column was then washed partially (≤ three column volumes) with 10 m*M* Tris pH 7.8, 0.05%(*w*/*v*) LDAO to remove a portion of the loosely bound components. Proteins were eluted with 10 m*M* Tris, 0.05%(*w*/*v*) LDAO, 100 m*M* imidazole pH 7.8 and subsequently desalted using a HiPrep 26/10 column (GE Heathcare). Collected fractions were combined and concentrated in a centrifugal filter (Amicon Ultra, 30 000 molecular-weight cutoff; Millipore, Billerica, Massachusetts, USA). The protein concentration and purity level were determined by UV–Vis–near-IR spectroscopy. The *A*_280_/*A*_800_ ratio was used to monitor the purity by assessing the amount of bacteriochlorophyll-containing RCs relative to the total protein content of the sample.
(ii) The sample of highest purity level (*A*_280_/*A*_800_ = 1.4) was prepared manually by passing the supernatant twice over a column composed of 10 ml Ni–NTA Superflow resin (Qiagen, Valencia, California, USA). The column was washed extensively with ten column volumes of 10 m*M* Tris pH 7.8, 0.05%(*w*/*v*) LDAO to remove loosely bound components. Proteins were eluted with 10 m*M* Tris, 0.05%(*w*/*v*) LDAO, 100 m*M* imidazole pH 7.8 and were subsequently additionally purified on a column of DEAE Sephacel resin (Sigma–Aldrich, St Louis, Missouri, USA). The column was washed with more than five column volumes of 10 m*M* Tris pH 7.8, 0.05%(*w*/*v*) LDAO until the *A*_280_ of the eluate was less than 0.1. Proteins were eluted with 10 m*M* Tris, 0.05%(*w*/*v*) LDAO, 280 m*M* NaCl pH 7.8 with manual collection of fractions. Fractions were combined and concentrated with a centrifugal filter as described above. The protein concentration was determined by UV–Vis–near-IR spectroscopy.

Samples of intermediate purity were prepared by simple mixing of the above two extremes and were monitored spectroscopically. All RC samples were concentrated to an *A*_800_ of 18 (∼6 mg ml^−1^).

### 2.2. Purity assessment of RC samples

The purity levels of *R. sphaeroides* RC samples were also assessed by SDS–PAGE to determine the nature of the protein contaminants. The gels were PAGEr Gold Precast 8–16% acrylamide (Lonza, Walkersville, Maryland, USA) or Novex NuPAGE bis-tris 4–12% acrylamide (Invitrogen, Carlsbad, California, USA) and were stained with Bio-Safe Coomassie Stain (Bio-Rad, Hercules, California, USA) or Coomassie Brilliant Blue R-250 according to the manufacturers’ protocols.

In addition, the lipid content of each sample was determined by thin-layer chromatography (TLC) as described previously (Eriks *et al.*, 2003 ▶; Kors *et al.*, 2009 ▶). In brief, a TLC tank lined with 3 mm CHR pure cellulose chromatography paper (Whatman, Florham Park, New Jersey, USA) and filled with solvent (chloroform:methanol:ammonium hydroxide, 63:35:5 by volume; Fisher Scientific, Waltham, Massachusetts, USA) was sealed and equilibrated for 1 h. Samples (5 µl) were spotted on 10 × 20 cm Silica Gel 60 TLC plates (EMD Chemicals, Gibbstown, New Jersey, USA). Spotted plates were allowed to dry and were then placed into the sealed TLC chamber. Once the solvent had migrated to 1–2 cm from the top of the plates, they were removed and allowed to dry thoroughly.

For lipid visualization, a large desiccator was heated to 333 K in a hot-water bath. Resublimed iodine crystals (Fisher Scientific) were placed at the bottom of the desiccator and the TLC plates were stained in the desiccator for no longer than 15 min. Plates were imaged immediately in order to record the maximum intensity of the short-lived iodine signal.

Destained gels and stained TLC plates were scanned and *ImageJ* v.1.36 (Abramoff *et al.*, 2004 ▶) was used to quantify the signals from the images.

### 2.3. Methods for crystallization of RC samples

Crystallization experiments were performed in parallel using LCP, plug-based microbatch and sitting-drop vapor-diffusion crystallization techniques and were conducted within 3 d of protein purification and initial characterization.

#### 2.3.1. Vapor diffusion

Sitting-drop vapor-diffusion crystallization trials of RC samples with varying *A*_280_/*A*_800_ ratios were set up similarly to previously described conditions (Chang *et al.*, 1985 ▶) at room temperature. Droplets consisted of 0.9–2.0 mg ml^−1^ RCs, 16–21%(*w*/*v*) PEG 4000, 0.28 *M* NaCl, 3%(*w*/*v*) 1,2,3-heptanetriol (high-melting point isomer; Sigma–Aldrich) and 0.05%(*w*/*v*) LDAO. Typical droplet volumes were 12.5–25 µl (for microlitre trials) or 400 nl (for nanolitre trials). This RC mixture was equilibrated against 0.75–1 ml reservoirs (for microlitre trials) or 150 µl reservoirs (for nanolitre trials) of 10 m*M* Tris pH 7.8, 0.56 *M* NaCl, 25%(*w*/*v*) PEG 4000. Plates were stored at room temperature in the dark. The microlitre-volume trials were set up manually, whereas the nanolitre-volume trials were set up using a robot (Mosquito, TTP LabTech, Melbourn, England). Independent control crystals of RC grown under the conditions referenced above grew up to 4 mm in size in large setups. Such crystal sizes agree with those reported in the literature (Chang *et al.*, 1985 ▶).

#### 2.3.2. Microbatch plugs

The batch-mode experimental setup has been described previously (Li *et al.*, 2006 ▶). On a microfluidic chip, the protein sample was combined with buffer and crystallization-inducing agent streams and 10–15 nl plugs were formed in the Teflon tubing (200 µm inner diameter, 250 µm outer diameter). The plugs were carried by a mixture of perfluoro-tri-*n*-butylamine and perfluoro-di-­*n*-­butylmethylamine (FC-40). The buffer was 0.15%(*w*/*v*) LDAO, 9.8%(*w*/*v*) 1,2,3-heptanetriol, 10 m*M* Tris pH 7.8; the crystallization-inducing agent was 0.15%(*w*/*v*) LDAO, 9.8%(*w*/*v*) 1,2,3-heptanetriol, 1.1 *M* NaCl, 50%(*w*/*v*) PEG 4000, 50 m*M* Tris pH 7.8. The flow rate of the protein sample was kept constant at 0.6 µl min^−1^ and the stream of the crystallization-inducing agent ranged from 0.4 to 0.7 µl min^−1^ with 0.1 µl min^−1^ increments; the buffer stream ranged from 0.4 to 0.1 µl min^−1^ accordingly to maintain the total aqueous flow rate at 1.4 µl min^−1^ and the carrier fluid, FC-40, ranged from 1.4 to 2.6 µl min^−1^ with 0.3 µl min^−1^ increments in phase with the increase in flow rate of the crystallization-inducing agent. By changing the flow rate of the carrier fluid, the size of the plugs changed, a parameter that can be used to index the concentration of crystallization-inducing agent such that larger plugs contain less of it (Li *et al.*, 2006 ▶). With this setup, the protein, LDAO and heptanetriol were kept at the same concentrations of 2.6 mg ml^−1^, 0.1%(*w*/*v*) and 5.6%(*w*/*v*), respectively; the NaCl and PEG 4000 concentrations ranged from 0.55 *M* and 25%(*w*/*v*) to 0.31 *M* and 14%(*w*/*v*), respectively. In order to prevent evaporation, the Teflon tubing housing the crystallization trials was stored in additional glass tubing (1 mm inner diameter, 2 mm outer diameter) which was pre-filled with perfluorotripentylamine (FC-70) and sealed with wax at both ends. The trials were incubated at 296 K.

#### 2.3.3. LCP

Proteo-LCP was prepared using a 40:60(*w*:*w*) ratio of protein solution:monoolein (Nu-Chek, Elysian, Minnesota, USA) by the microcrystallization cubic phase method (Nollert, 2004 ▶). Briefly, one 250 µl syringe containing pre-weighed solid monoolein (MO) was connected to a second 250 µl syringe containing protein solution and the solutions were passed back and forth *via* a syringe coupler (Cubic LCP kit; Emerald BioSystems, Bainbridge Island, Washington, USA) until the material became transparent and uniform. A 10 µl syringe mounted in a repeating dispenser (Hamilton, Reno, Nevada, USA) was loaded with proteo-LCP by way of the syringe coupler. Proteo-LCP slugs of 0.4 µl volume were delivered into the drop chamber of a Compact Jr plate (Emerald BioSystems) which was pre-dispensed with 2 µl crystallization-inducing reagent solution from the reservoir. After sealing with tape, the drop chamber was equilibrated with a reservoir of 80 µl in volume. The plates were wrapped in foil and stored in a dark cabinet at 289 K. Samples in LCP were incubated in a series of conditions (comprising a 48-­condition grid matrix) in which the concentration of Jeffamine M-600 (Sigma–Aldrich) was varied from 7 to 18%(*v*/*v*), the ammonium sulfate (Sigma–Aldrich) concentration ranged from 0.7 to 1.15 *M* and the buffer (HEPES pH 7.5) was held constant at 1 *M*. A significant variation was observed in the pH of Jeffamine M-600 obtained from different suppliers and in different lots obtained from the same supplier; care was therefore taken to make certain that the crystallization mixtures had a final measured pH of 7.2. Statistics for LCP crystallization-trial success were computed as a percentage of hits from a 48-­condition screen.

### 2.4. X-ray diffraction of RC crystals

In order to minimize the potential damage to crystals arising from handling and cryoprotection, crystals from LCP and vapor-diffusion trials were examined at room temperature without transfer from the bulk crystallization solution. As expected, the X-ray diffraction limits of RC crystals tested at room temperature were poor (∼10 Å). Crystals of RCs grown for control purposes and tested for X-ray diffraction at liquid-nitrogen temperature routinely diffracted to better than 3.5 Å resolution using synchrotron radiation. Large portions of the crystallization drop were drawn into glass capillaries by gentle aspiration of bulk crystallization-inducing reagent. Capillaries containing crystals were mounted on a goniostat and aligned in the X-ray beam using a microscope. In the case of microbatch trials, *in situ* diffraction experiments were conducted. X-ray diffraction experiments were conducted on beamline 19BM of the Advanced Photon Source. Crystals were exposed to the unattenuated beam for 5 s and diffraction data were collected on a CCD detector to assess the intrinsic diffraction limit.

### 2.5. Lipids from *R. sphaeroides*, brain and *E. coli*

For the preparation of *R. sphaeroides* lipid samples, approximately 4 g of the membrane-debris pellet from §[Sec sec2.1]2.1 was homogenized in a final volume of 8 ml 10 m*M* Tris pH 7.5. This membrane solution was used as the starting material in an organic lipid-extraction pro­cedure (Bligh & Dyer, 1959 ▶) comprising serial additions, with vortexing, to the homogenate of 30 ml 1:2 CHCl_3_:MeOH, 10 ml CHCl_3_ and 10 ml deionized water. The solution was centrifuged for 5 min at 1000*g* at room temperature to separate the phases. The lower organic phase was transferred to a clean glass vial, dried under vacuum and stored at 253 K under inert gas. Polar brain (porcine) lipid extract and *E. coli* polar lipid extract were purchased from Avanti Polar Lipids (Alabaster, AL, USA).

### 2.6. LCP crystallization in the presence of lipids

Polar brain, polar *E. coli* and extracted *R. sphaeroides* lipids were reconstituted independently to a concentration of 100 mg ml^−1^ in a MeOH–CHCl_3_ mixture (1:2 ratio). Parallel experiments were conducted by replacing 0.5–30% of the mass of MO (0.3–18% of the total LCP mass) in LCP trials with one of the above lipid mixtures and setting up trials as described in §[Sec sec2.3.3]2.3.3. The MO–lipid material was preformed by melting at 310 K to facilitate mixing. The protein sample used was the *R. sphaeroides* RC sample of highest purity (*A*_280_/*A*_800_ = 1.4). The resultant LCP preparations were screened for crystallization against the same 48-condition crystallization screen employed previously (§[Sec sec2.3.3]2.3.3).

### 2.7. Preparation of *R. sphaeroides* membrane samples

Pellets containing *R. sphaeroides* membrane vesicles (§[Sec sec2.1]2.1) were resuspended in 12.5 ml buffer 1 per gram and were treated with 0.03%(*v*/*v*) LDAO for 15 min with stirring in the dark. This low concentration of LDAO preserved the embedded membrane proteins but permeabilized the inside-out vesicles, releasing the trapped soluble contents (periplasmic proteins). The detergent was removed by ultracentrifugation at 245 000*g* for 120 min at 277 K. The pelleted membranes were homogenized in buffer 1 and were treated in the same manner two more times; they were then resuspended in buffer 1.

### 2.8. LCP crystallization in the presence of membrane fragments

Purified membranes from *R. sphaeroides* (§[Sec sec2.7]2.7) were added to the LCP-based crystallization trials by diluting membranes into the aqueous protein fraction prior to mixing it with solid MO. Membrane paste was added by replacing 1–30%(*w*/*v*) of the purified LDAO-solubilized *R. sphaeroides* RCs (0.4–12% of the total mass of the LCP trial). The protein sample used was an *R. sphaeroides* RC sample of intermediate purity (*A*_280_/*A*_800_ = 2.0). The resulting LCP preparations were screened for crystallization against the same 48-condition crystallization screen employed previously (§[Sec sec2.3.3]2.3.3).

### 2.9. Characterization of protein samples

**Figure 1:**
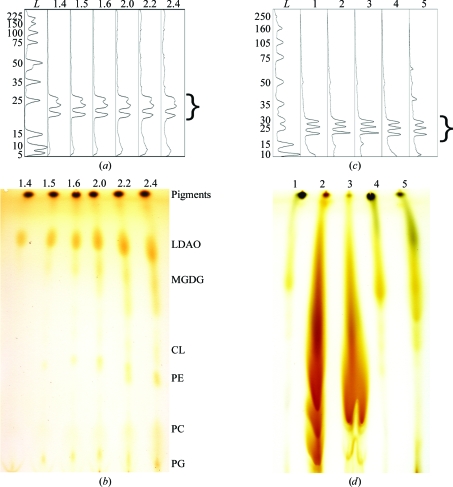
Characterization of protein and lipid impurities found within partially purified RC samples used for crystallization experiments. Protein analyses (*a*, *c*) are depicted as digitized intensities from Coomassie-stained SDS–PAGE gels. Lipid analyses (*b*, *d*) comprise iodine-stained TLC plates. For the partially purified samples (*a*) and (*b*), the RC content was kept constant using 12 and 30 µg in each gel and TLC lane, respectively. The numbers above the lanes indicate the *A*_280_/*A*_800_ ratio of the sample. Assignments of spots on the TLC plate in (*b*) signify the detergent and lipid components present in the samples that are resolved by this solvent system: Pigments, a mixture comprised of bacteriochlorophylls, bacteriopheophytins, carotenoids and quinones; LDAO, *N*,*N*-dimethyl­dodecylamine-*N*-oxide; MGDG, monogalactosyldiacylglycerol; CL, cardiolipin; PE, phosphatidyl­ethanolamine; PC, phosphatidylcholine; PG, phosphatidylglycerol. Increases in LDAO intensities observed on the TLC plate in (*b*) can be attributed to an overall increase in total protein in samples with decreasing purity (increasing levels of impurities), resulting in a higher overall level of PDCs (daCosta & Baenziger, 2003 ▶). For samples with lipids or membranes added (*c*, *d*), the exact contents of the LCP-based trials (0.5 mg of each) in the absence of MO were loaded for analysis. The samples contained (1) RCs only, (2) RCs plus 12% polar brain lipids, (3) RCs plus 18% polar *E. coli* lipids, (4) RCs plus 1.2% extracted *R. sphaeroides* lipids, (5) RCs plus 12% *R. sphaeroides* whole membranes. For both sets of samples, bands corresponding to the three protein subunits (L, M and H; ∼25–30 kDa) of the *R. sphaeroides* RC complex are marked with a bracket and the lanes containing molecular-weight standards [ProSieve Protein Markers from Lonza in (*a*) and Full-Range Rainbow Marker from GE Healthcare in (*c*)] are indicated (lane *L*).

The purest preparations of *R. sphaeroides* RCs that retained all aspects of their light-driven charge-separation function were characterized by *A*_280_/*A*_800_ ratios (total protein/bound monomeric bacteriochlorophyll) of 1.2. SDS–PAGE gels of these ultrapure RCs (stained with Coomassie Blue or Silver) revealed few if any impurities and such a sample was defined in this study as being 100% pure. Purified RC samples having absorption ratios of ≤0.4 (∼85% pure) are known to be highly crystallizable (Pokkuluri *et al.*, 2002 ▶). To explore the limits of such samples with various crystallization approaches, two types of RC samples were purified from membranes of *R. sphaeroides* (*A*_280_/*A*_800_ = 1.4 and 2.4). Samples of intermediate purity (*A*_280_/*A*_800_ = 1.5, 1.6, 2.0 and 2.2) resulted by simple linear mixing. Analysis by UV–Vis–near-IR absorption spectroscopy, complemented by SDS–PAGE (Fig. 1 ▶ *a*), where band intensities were quantified by *ImageJ*, suggested that these RC samples ranged in purity from 86 to 50%, respectively. This implies that 14–50% of the samples were contaminating proteins. As expected, a gradual increase in background staining was observed on gels for samples with increasing *A*_280_/*A*_800_ ratios and was the direct result of rising contamination levels of proteins of various sizes.

Gradual increases in protein contamination were also accompanied by increased levels of lipids that were remnants of the suboptimal purification process. TLC analysis was used to visualize the general lipid content (Fig. 1 ▶ *b*). Not surprisingly, background staining at specific *R*_f_ values attributable to lipids in the sample increased with increasing *A*_280_/*A*_800_ ratio. The RC sample of least purity (*A*_280_/*A*_800_ = 2.4) appeared to contain 12 times more iodine-staining material as quantified using *ImageJ* in comparison to the sample with the highest purity level (*A*_280_/*A*_800_ = 1.4). The most intensely staining lipid species were identified based upon their known relative mobilities in TLC, their presence in purified RC samples and their ability to be removed during purification if certain chromatographic schemes were utilized (Albuquerque *et al.*, 2002 ▶; Catucci *et al.*, 2004 ▶; Dezi *et al.*, 2007 ▶; Ventrella *et al.*, 2007 ▶; Camara-Artigas *et al.*, 2002 ▶).

The impurities that were encountered in these samples would be similar to the impurities encountered in any purified membrane-protein sample, especially for a protein that has not been characterized and that has possibly been purified for the first time. No purification method is immune to the presence of contaminants. Even with recent advances in affinity chromatography, the purification of membrane-protein samples to homogeneity is frequently an overwhelming obstacle in structural and functional studies. Difficulties in membrane-protein purification are in many ways inherent to the requirement for using a detergent to solubilize the macromolecules, thereby making them amenable to aqueous-based chromatographies. Thus, when purifying membrane proteins, one is actually purifying a protein–detergent complex, with which endogenous lipids frequently associate and copurify. The detergent micelle that encompasses the hydrophobic regions of the proteins limits access to tags in affinity chromatography, masks interactions with charged resins in ion-exchange chromatography and modulates size and limits separation in size-exclusion chromatography. It is for these reasons that we sought a crystallization approach that was tolerant of moieties that are encountered commonly in membrane-protein samples.

### 2.10. Characterization of crystallization additives

The RC samples with varying amounts of added lipid (polar brain lipids, polar *E. coli* lipids or extracted *R. sphaeroides* lipids) were analyzed by SDS–PAGE (Fig. 1 ▶ *c*). From these analyses, it was determined that none of the lipid extracts utilized contained extraneous protein, as reflected by the relatively constant background:RC ratios, even when large quantities (18% of the total weight of the droplet) of lipids were added.

Likewise, the quality of the lipid extract added to each RC sample was assessed on TLC plates (Fig. 1 ▶ *d*). Background staining attributable to a mixture of lipids in the samples increased with increasing amounts of added lipid. The samples shown represent those with the highest percentage for each lipid additive utilized in these experiments. Although individual species cannot be resolved, it is clear that there are differences in the numbers and types of lipids. The samples containing polar brain lipids appear to be the most diverse, the samples containing the extracted *R. sphaeroides* lipids are the least complex and the polar *E. coli* lipid extract may share species with the polar brain lipids.

## 3. Results and discussion

### 3.1. Effect of protein purity on crystallization

**Figure 2:**
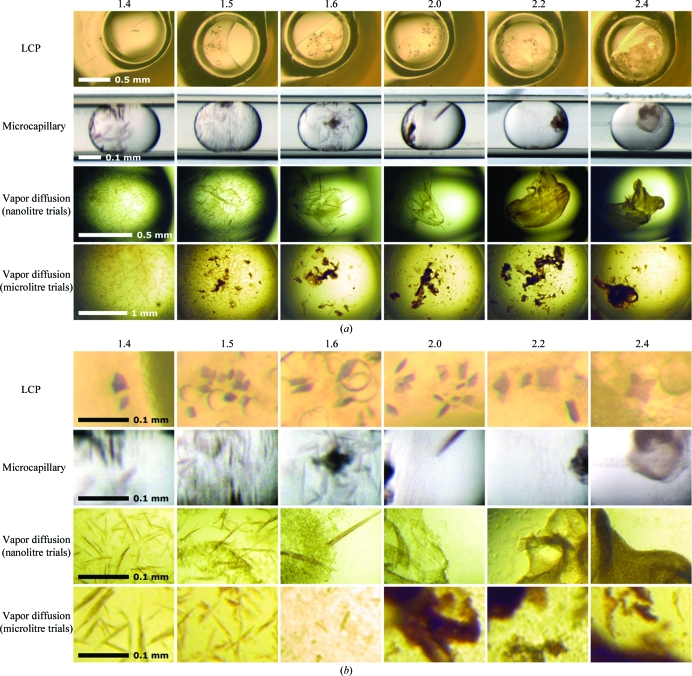
Images of crystallization experiments with RC samples of increasing purity (as reflected by the *A*_280_/*A*_800_ ratio) using LCP, microfluidics or sitting-drop vapor-diffusion techniques. (*a*) Holistic view of crystallization trials and (*b*) enhanced magnification, to a uniform scale, for comparison of crystal size and quality.

When compared with sitting-drop vapor diffusion and crystallization in the plug-based microbatch system, our results clearly show that the LCP method produced RC crystals from samples containing the highest contamination levels (Table 1 ▶, Fig. 2 ▶). Importantly, the contaminants faithfully represent native protein and lipid impurities that typically arise from incomplete purification processes. Should this finding hold true for many membrane proteins, it would be advisable for membrane-protein researchers to focus on LCP-based crystallization experiments early on in membrane-protein purification and crystallization trials.

RC crystals grew best in the LCP trials and are almost unaffected by impurities, as shown in Fig. 2 ▶. Indeed, the LCP method tolerated levels of impurities that were equal to the amount of target protein present (up to 50% impurities; Table 1 ▶, Fig. 1 ▶). Crystals appeared in setups with RC *A*_280_/*A*_800_ absorption ranging from 1.4 to 2.4 (Fig. 2 ▶) and were visible after 48 h; larger crystals grew to full size after 5 d. In subsequent experiments with an RC sample of *A*_280_/*A*_800_ = 2.8 (∼43% RC) no crystals were obtained (data not shown).

For vapor-diffusion trials the formation of crystals was limited to an *A*_280_/*A*_800_ ratio of 1.6 or below (∼75% RC), while microbatch trials produced few crystals at an *A*_280_/*A*_800_ ratio of 2.0 (∼60% RC; Table 1 ▶, Fig. 1 ▶).

For nanolitre sitting-drop vapor-diffusion trials, samples with lower impurity levels (A_280_/*A*_800_ ratios up to 1.6) yielded crystals within 2–3 d. Samples with *A*_280_/*A*_800_ ratios of 1.4 and 1.5 yielded crystals within 2–3 d for microlitre trials, while crystals started to appear in a sample with an *A*_280_/*A*_800_ ratio of 1.6 after ∼10 d (Fig. 2 ▶). Crystals were never observed with samples having *A*_280_/*A*_800_ ratios of 2.0, 2.2 or 2.4 using vapor diffusion. The tolerance for protein impurities using vapor-diffusion approaches was independent of the volume of the experiment.

For microfluidic plug-based batch trials, samples with *A*_280_/*A*_800_ ratios of up to 1.6 yielded crystals within 2–3 d. After 10 d, the sample with an *A*_280_/*A*_800_ ratio of 2.0 also yielded crystals. Crystals were never obtained from samples with *A*_280_/*A*_800_ ratios of 2.2 or 2.4 using this approach (Fig. 2 ▶).

**Figure 3:**
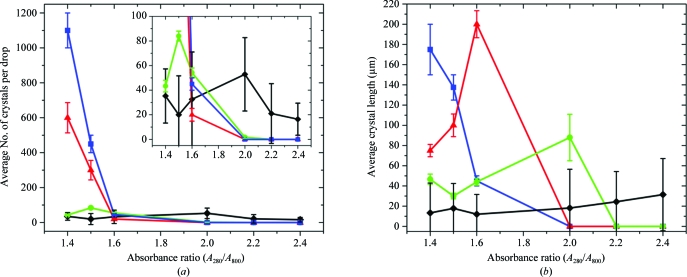
The effect of impurities (variations in the *A*_280_/*A*_800_ ratio) on (*a*) the average number of crystals obtained and (*b*) the average crystal length in LCP (black diamonds), microfluidics (green circles), nanolitre vapor-diffusion (red triangles) and microlitre vapor-diffusion (blue squares) trials. The inset in (*a*) represents a magnified view of the portion of the graph in which the data for LCP and microfluidic trials reside. Error bars represent standard deviations from three or more independent crystallization experiments.

For vapor-diffusion and microbatch plug-based crystallizations that employed PEG 4000 as the crystallization-inducing agent, the numbers and sizes of crystals scaled with the volume of the crystallization droplet (Fig. 3 ▶). In addition, the number of crystals obtained decreased rapidly as the impurity level increased. For microlitre-volume sitting-drop vapor-diffusion trials crystal size decreased rapidly with the addition of impurities, while crystal size became maximal at intermediate impurity levels (presumably owing to decreased nucleation events) for nanolitre-volume vapor-diffusion and nanolitre-volume microbatch trials (Fig. 3 ▶).

These results demonstrate that crystal nucleation, not crystal growth, is affected by the presence of protein impurities. Crystal size was limited in the samples of highest purity presumably because of the large number of nuclei that formed, which ultimately limited the amount of protein available for crystal growth. Although the number of nuclei decreased in samples of lesser purity, crystal growth was not impeded as these samples produced larger crystals than those experiments in which showers of crystals appeared.

**Figure 4:**
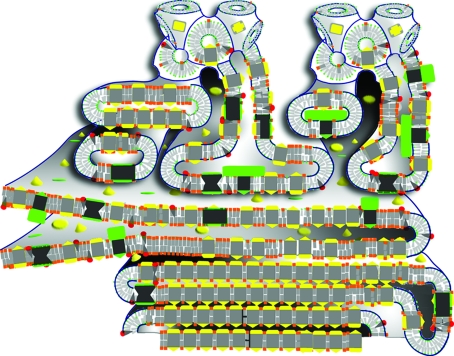
Illustration depicting why the LCP methodology has a high tolerance for contaminating protein species (black and green), based on the quantitative mechanistic framework for crystal growth (Grabe *et al.*, 2003 ▶; Nollert *et al.*, 2001 ▶). Here, impurities are excluded from the crystal-growth process, essentially providing a microenvironment enriched in the crystallizing species (gray and yellow), thus favoring crystal growth. Along the lines of a ‘kinetic exclusion mechanism’, contaminating protein species with large hydrophilic or hydrophobic moieties face an energy penalty for diffusion in curved membranes with small channels, resulting in less unproductive aggregation and hence less interference with the desired crystallization process. Similarly, lipidic cubic phases form substantial diffusion barriers for soluble proteins (Razumas *et al.*, 1996 ▶), trapping soluble contaminating proteins within the small hydrophilic channels of the LCP matrix, where they are excluded from poisoning the crystal-growth surface. The local absence of contaminating species allows crystals to grow as they would in solution-based crystallization approaches (batch and vapor diffusion) using samples of higher purity.

For the LCP-based crystallizations utilizing Jeffamine and ammonium sulfate as the crystallization-inducing agents, the number of crystals obtained remained constant across the protein purity levels (Fig. 3 ▶). Not only did nuclei formation seem to be less impeded for the LCP method, but the sizes of the crystals obtained using this approach actually increased with decreasing protein purity. This surprising result violates the understanding of typical crystal growth in aqueous solution, where impurities disrupt the uniform assembly of layers of the crystal lattice (Yip & Ward, 1996 ▶). The microscopic distribution of impurities in the lipid mesophase matrix may allow more efficient diffusion of monodispersed RCs to the area of crystal growth and efficient diffusion of impurities away from the zone of crystal growth. Crystal growth may have ultimately been limited by diffusion and the complex nature of the lipid mesophase matrix (Fig. 4 ▶).

We also noted that increasing sample-impurity levels resulted in a greater amount of precipitate in microbatch trials as well as in microlitre-volume and nanolitre-volume vapor-diffusion trials (Fig. 2 ▶). This gross aggregation formed with kinetics similar to the formation of crystals and was not observed in LCP crystallization trials (Fig. 2 ▶). The presence of contaminating proteins and lipids obviously contributed to this unproductive end product, impeding structured crystal formation from input proteins that are crystallizable in purer form. Although the lack of this aggregation in the lipid mesophase may be a consequence of the differences in crystallization-inducing agents, its absence in the lipid mesophase may help to explain the improved tolerance of this method towards the impurities that were introduced (Fig. 4 ▶). It is possible that the lipid bilayer in the lipid mesophase may prevent aggregation or crystal-growth poisoning by solubilizing impurities.

### 3.2. Effect of protein purity on crystal quality

**Figure 5:**
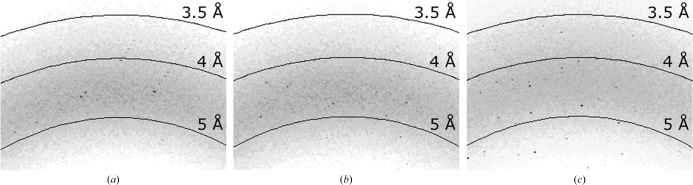
Diffraction images of crystals produced *via* LCP approaches using RC samples of varying purity, as reflected by the *A*_280_/*A*_800_ ratio. The diffraction limit at room temperature of the three crystals shown with *A*_280_/*A*_800_ ratios of (*a*) 1.4, (*b*) 1.6 and (*c*) 2.4 were determined to be 3.56, 3.58 and 3.48 Å, respectively, using routines built into the software package *HKL*-2000 (HKL Research Inc., Charlottesville, Virginia, USA).

RC crystals grew in the LCP trials and diffracted X-rays to a resolution of ∼3.5 Å regardless of the purity level in the crystallization setups (Fig. 5 ▶). Hence, the crystallization behavior and crystal quality are tolerant to impurity levels typically found after only one or two purification steps. The latter is a highly desirable attribute of this crystallization approach. This finding is reminiscent of the crystallization of bacteriorhodopsin from purple mem­brane fractions that have undergone no chromatographic purification (Nollert *et al.*, 1999 ▶) and may indicate a general feature of the LCP-based membrane-protein crystallization method to yield crystals of sufficient X-­ray diffraction quality from relatively impure starting material.

Conversely, crystals grown by vapor diffusion and microbatch techniques did not diffract beyond 10 Å resolution with synchrotron radiation. A factor in this very low diffraction resolution may have been that the needles that formed had only one dimension larger than 10 µm compared with the ∼100 µm^2^ size of the X-ray beam. Larger cryopreserved crystals grown previously using slight variations in crystallization con­ditions have diffracted to 3.2 Å resolution (Chang *et al.*, 1991 ▶; Marone *et al.*, 1999 ▶) and we presume that the quality of the crystals that formed in these experiments is similar.

In this study, the X-ray diffraction limits of the small crystals formed from impure membrane-protein samples equalled the diffraction limits of larger crystals formed by the same method but starting from pure protein. If this observation can be generalized, the diffraction limits of crystals obtained from initial LCP-based trials should predict the quality of larger crystals that one could expect to obtain from samples generated from optimized purification protocols.

### 3.3. Effect of lipids and membranes on LCP-based crystallization

**Figure 6:**
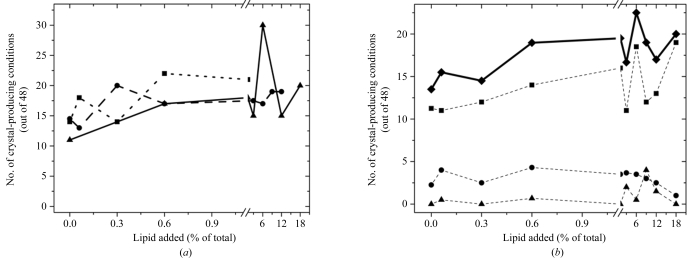
The effect of the addition of lipids on LCP crystallization of RCs. (*a*) The number of conditions producing crystals out of a total of 48 from the ammonium sulfate and Jeffamine grid screen, as a function of increasing amounts of extracted *R. sphaeroides* lipids (squares, dotted line), polar *E. coli* lipids (triangles, unbroken line) and polar brain lipid (circles, dashed line). (*b*) The quality of the RC crystals produced in LCP trials as a function of increasing amounts of lipid additives. Data for extracted *R. sphaeroides*, polar *E. coli* and polar brain lipids were averaged. Values plotted represent either the total number of crystals observed (diamonds, unbroken line) or a high-quality (triangles, dotted line), medium-quality (circles, dotted line) or low-quality (squares, dotted line) visual score based on the size, degree of symmetry and edge definition of the crystals obtained.

Since the formation of RC crystals in LCP-based crystallization trials was robust when challenged with non-RC protein, we tested whether the same holds for lipids. In this regard, the data show that the amount and source (polar brain, polar *E. coli* or extracted *R. sphaeroides* lipids) of the lipids added to the LCP-based crystallization experiments had no negative effect on crystallization success (Fig. 6 ▶ *a*). The number of crystal-producing conditions was similar for all three lipid sources and slight increases were observed in the number of crystal-producing conditions containing more than 5% lipid, with an average of two additional crystal conditions relative to those experiments containing less than 5% lipid (Fig. 6 ▶ *a*).

Surprisingly, adding extra lipids to LCP trials generally increased the total number of conditions that produced crystals. However, these experiments were dominated by conditions producing crystals of relatively low quality (Fig. 6 ▶ *b*). The number of conditions producing medium-quality crystals remained constant, while the number of conditions reporting at least one high-quality crystal, even though relatively low, increased significantly with the addition of lipid. This increase was most pronounced at concentrations of 5–20% added lipids (Fig. 6 ▶ *b*).

Hence, none of the lipid mixtures inhibited crystal growth. On the contrary, the use of endogenous *R. sphaeroides* lipids in the formation of LCP led to slightly more hits per screen on average (Fig. 6 ▶).

**Figure 7:**
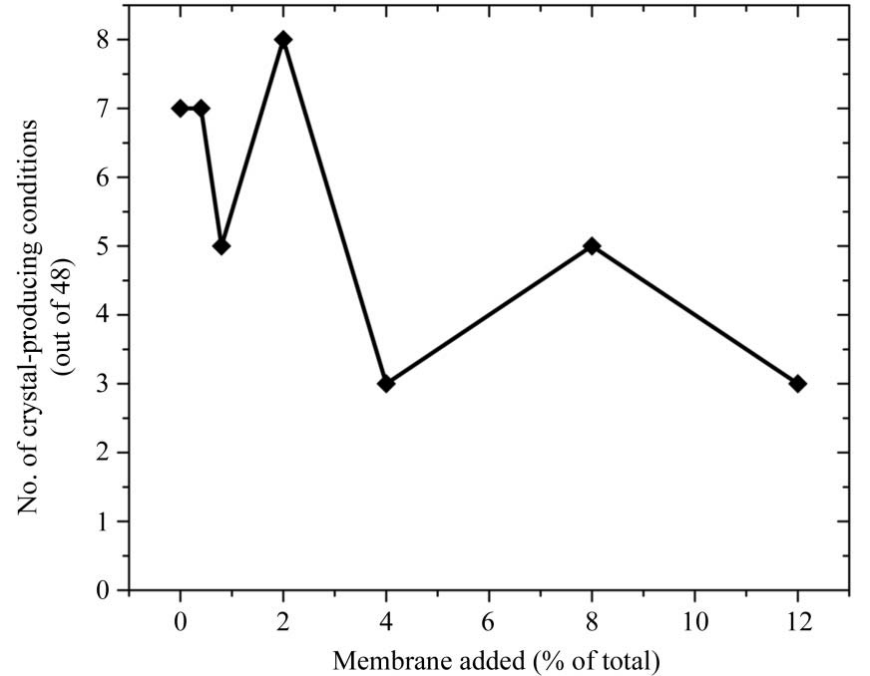
The effect of membrane additives (intact vesicles from engineered RC-deletion strains of *R. sphaeroides*) on the crystallization of RC samples using LCP approaches. The data plotted represent the number of conditions producing crystals out of a total of 48 from the ammonium sulfate and Jeffamine grid screen.

Since the replacement of up to 30% of the MO in LCP preparations (18% of the total weight of the droplet) with lipid extracts did not have a negative impact on the crystallization of *R. sphaeroides* RCs, the effect of adding RC-depleted membranes to LCP preparations prior to crystallization was examined (Fig. 7 ▶). Again, these additions had a minimal impact on crystallization, with the number of crystal-producing conditions decreasing only slightly as intact membranes were introduced (Fig. 7 ▶). Unexpectedly, a significant number of conditions still produced crystals even when 30%(*w*/*v*) of the sample consisted of *R. sphaeroides* membranes, showing that additions of intact membranes (lipids plus protein) were also tolerated well by the LCP-based crystallization approach.

The observed robustness of the LCP-based crystallizations are in line with a report on the crystallization of bacterio­rhodopsin in the absence of detergent starting out from the enriched purple membranes of *Halobacterium salinarum* (Nollert *et al.*, 1999 ▶). We may rationalize these findings by proposing that the doping of LCP-based crystallization experiments with detergents, protein, lipids or membranes may encourage lipid membrane restructuring or enhance mem­brane transitions during the crystallization process (Nollert *et al.*, 2001 ▶; Fig. 4 ▶).

## 4. Concluding remarks and perspectives

In order to provide guidance for the experimental design of initial membrane-protein crystallization trials, we have undertaken a series of comparative experiments to explore the effect of impurities in the form of proteins and lipids on the crystallization of membrane proteins in vapor-diffusion, in plug-based microbatch and in LCP-based crystallization trials. Employing the bacterial photosynthetic RC from *R. sphaeroides* as a model system, we found that its crystallization using the LCP approach tolerated the highest levels of both protein and lipid contaminants. If this finding holds true for many membrane proteins then it would be advisable to include the LCP methodology in the first set of crystallization trials. Alternatively, LCP-based crystallization trials should be employed with membrane-protein samples that show sub­stantial levels of impurities. Hence, as a practical matter, membrane-protein purification development efforts may be best accompanied by LCP-based crystallizations and approaches which allow relatively impure fractions to be used in structural studies, as these samples have been shown to yield good starting points for the optimization of purification and crystallization experiments.

## Figures and Tables

**Table 1:** The abilities of the various crystallization methods to tolerate increased levels of impurities

Crystallization method	Maximum *A*_280_/*A*_800_ ratio of sample producing crystals	RC content (%)	Impurity content (%)
LCP	2.4	50	50
Microcapillary	2.0	60	40
Sitting-drop vapor diffusion (nanolitre trials)	1.6	75	25
Sitting-drop vapor diffusion (microlitre trials)	1.6	75	25
